# Impact of body mass index on clinical presentation and prognosis in myasthenia gravis

**DOI:** 10.1186/s13023-025-03902-1

**Published:** 2025-07-14

**Authors:** Hong-xi Chen, Zi-ya Wang, Na-na Zhang, Xue Lin, Zi-yan Shi, Xiao-fei Wang, Ying Zhang, Qin Du, Ling-yao Kong, Dong-ren Sun, Rui Wang, Yang-yang Zhang, Shuang-jie Li, Yu-wei Da, Hui-yu Feng, Hong-yu Zhou

**Affiliations:** 1 Department of Neurology, West China Hospital, Sichuan University, Guo Xue xiang #37, Chengdu, 610041 China; 2 Department of Neurology, Xuanwu Hospital, Capital Medical University, Beijing, China; 3 Department of Neurology, The First Affiliated Hospital, Sun Yat-sen University, Guangzhou, China

**Keywords:** Body mass index, Myasthenia Gravis, Generalization of ocular myasthenia Gravis, Activities of daily living response, Minimal symptom expression

## Abstract

**Background:**

The literature lacks consistent information on the correlation between baseline body mass index (BMI), clinical presentation, and prognosis in patients with myasthenia gravis (MG). This observational multicenter prospective cohort study included patients with MG from February 2017 to June 2023, categorizing them by baseline BMI. The primary outcome was the time to generalization of ocular MG. Secondary outcomes included the time to Activities of Daily Living (ADL) response and Minimal Symptom Expression (MSE). Kaplan-Meier curves and multivariable Cox proportional hazards regression models were used to assess the impact of BMI on these outcomes.

**Results:**

Out of 940 MG patients (510 women) included, 524 had a low BMI and 416 had a high BMI, with a median age of 50.00 years. Patients in the high BMI group were significantly older (*p* < 0.001), had a lower percentage of females (*p* < 0.001), and had a shorter disease duration (*p* = 0.014) compared to those with a low BMI. They also had higher rates of ocular onset (*p* < 0.001), ocular MG classification (*p* = 0.001), and acetylcholine receptor antibody seropositivity (*p* = 0.007), but a lower incidence of thymectomy (*p* = 0.027). During a median follow-up of 33.00 months, the adjusted Cox models revealed that a higher baseline BMI was associated with an increased risk of ocular MG generalization (HR 1.06; 95% CI 1.01–1.11; *p* = 0.026), but not with ADL response (HR 0.99; 95% CI 0.95–1.04; *p* = 0.779) or MSE (HR 0.97; 95% CI 0.92–1.02; *p* = 0.240).

**Conclusions:**

A higher baseline BMI was associated with an increased risk of ocular MG generalization but not with ADL response or MSE.

## Background

Myasthenia gravis (MG) is a condition where the immune system attacks the neuromuscular junctions, leading to muscle weakness and fatigue. This autoimmune disease mainly involves autoantibodies attacking the acetylcholine receptor (AChR) and muscle-specific kinase (MUSK). Symptoms of MG can vary from weakness in the eyes to generalized muscle weakness, and in severe cases, it can even lead to difficulties in swallowing or breathing [1]. It’s essential to identify factors that could worsen outcomes in MG patients and prevent the progression from ocular MG (OMG) to generalized MG (GMG). A multicenter retrospective cohort study found that the likelihood of OMG generalization was higher in patients with adult-onset OMG, abnormal repetitive nerve stimulation results, seropositivity for anti-AChR antibodies, and thymoma [2]. Furthermore, the immunotherapies can prevent generalization in OMG patients [3]. 

Recent studies have suggested that obesity is linked to a chronic inflammatory state characterized by elevated levels of inflammatory markers like leptin and C-reactive protein, as well as an imbalance in Th17/Treg ratio in adipose tissue, potentially leading to immune disorders [4, 5]. High body mass index (BMI) has been associated with the onset and severity of several autoimmune conditions, including neuromyelitis optica spectrum disorder, sarcoidosis, and dermatitis herpetiformis [6, 7]. 

In MG, there is an increase in Th17 cells and a decrease in Treg cells, creating a Th17/Treg imbalance that plays a crucial role in the disease progression and can be modulated through immunotherapy [8]. Based on this, we hypothesize that obesity-induced increases in Th17 cells and decreases in Treg cells could negatively affect MG and potentially worsen its clinical course. However, the relationship between BMI and MG appears inconsistent. A recent research suggests that a higher BMI might indicate better short-term outcomes [9], while other studies indicate that it could be an independent risk factor for post-thymectomy crisis, longer hospital stays, pregnancy-related complications, and reduced quality of life [10–13]. Due to the variability in results and the limited sample sizes of previous studies, a large-scale observational study is necessary to clarify the role of BMI in MG patients. Our study aims to explore the effects of baseline BMI on clinical presentation and prognosis in MG.

## Methods

### Study design and patients

This observational multicenter cohort study was conducted from February 2017 to June 2023. We prospectively included patients diagnosed with MG during their first visit to West China Hospital of Sichuan University, Xuanwu Hospital of Capital Medical University, and The First Affiliated Hospital of Sun Yat-sen University, representing diverse administrative regions of China. The diagnosis of MG was primarily based on typical clinical manifestations, including partial or generalized striated muscle fatigue that worsened after exercise and improved with rest. Additionally, the final diagnosis of MG required at least one of the following: (1) a positive serologic test for autoantibodies (AChR or MUSK antibodies), (2) a positive neostigmine test, or (3) abnormal repetitive nerve stimulation [1, 14]. Patients under 18 years of age and those without available BMI values were excluded. To eliminate the effect of medications on BMI, we also excluded patients who had used glucocorticoids (GCs) before baseline. All patients were regularly followed up through face-to-face or telephone consultations every 3 to 6 months.

### Data collection

We prospectively collected data on demographic information, including age, sex, and BMI at baseline, as well as clinical data. The clinical data included: (1) symptoms at onset (ocular or generalized), (2) classifications (ocular or generalized) according to the Myasthenia Gravis Foundation of America (MGFA), (3) disease duration (from the patient-reported initial onset of myasthenic symptoms to the time of baseline enrollment), (4) anti-AChR-IgG and anti-MUSK-IgG serology tested by commercial radioimmunoassay kit, enzyme-linked immunosorbent assay, or cell-based assay, (5) presence of thymoma, (6) thymectomy status, (7) treatment details, and (8) disease severity assessed using MG Activities of Daily Living (ADL) scores (patient-reported and physician-recorded) [15].

BMI was calculated as weight in kilograms divided by height in meters squared (kg/m²). According to the Chinese criterion (WS/T 428–2013) [16] patients were classified into two groups based on their baseline BMI: low BMI (BMI < 24 kg/m²) and high BMI (BMI ≥ 24 kg/m²). Thymoma was determined by pathological examination post-thymectomy or chest CT results. Patients received immunotherapy based on the individualized discretion of their treating physicians, which included GCs and non-GC immunotherapies (such as tacrolimus, azathioprine, and mycophenolate mofetil, cyclosporine, cyclophosphamide, rituximab, plasma exchange and intravenous immunoglobulin).

### Outcome measures

We compared the baseline clinical characteristics of patients between different BMI groups. In the follow-up study, the primary outcome was the time to generalization, defined as the interval from baseline OMG to the onset of signs or symptoms of GMG, including weakness in the limbs, face, bulbar region, neck, and respiratory muscles. The two secondary outcomes were assessed for patients with a baseline ADL score of ≥ 5: (1) the time to ADL response, defined as achieving at least a 2-point improvement in ADL score, and (2) the time to Minimal Symptom Expression (MSE), defined as achieving an MG-ADL score of 0 or 1 [17, 18].

### Statistical analysis

Continuous variables were presented as medians (interquartile ranges [IQRs]), and categorical variables were expressed as numbers (percentages). To compare baseline characteristics between the low BMI and high BMI groups, the Kruskal-Wallis rank sum test was used for continuous variables, while Pearson’s chi-squared test (χ2) was employed for categorical variables. Kaplan–Meier curves and log-rank tests were utilized to illustrate cumulative generalization of OMG, ADL response, and MSE rates between the low and high BMI groups. Variables with *p*-values < 0.05 in the comparisons of baseline characteristics, along with those recognized as risk factors in the literature, were included in multivariable Cox proportional hazards regression models to assess the risk of primary and secondary outcomes associated with different BMI values. This analysis estimated hazard ratios (HRs) and 95% confidence intervals (95% CIs). The follow-up duration in the survival analyses was defined as the period from baseline to the occurrence of the outcomes or the last follow-up before the study ended. Statistical significance was set at *p* < 0.05. All analyses were conducted using the R statistical software package (version 4.1.2; R Foundation for Statistical Computing, Vienna, Austria).

## Results

**Fig. 1:**
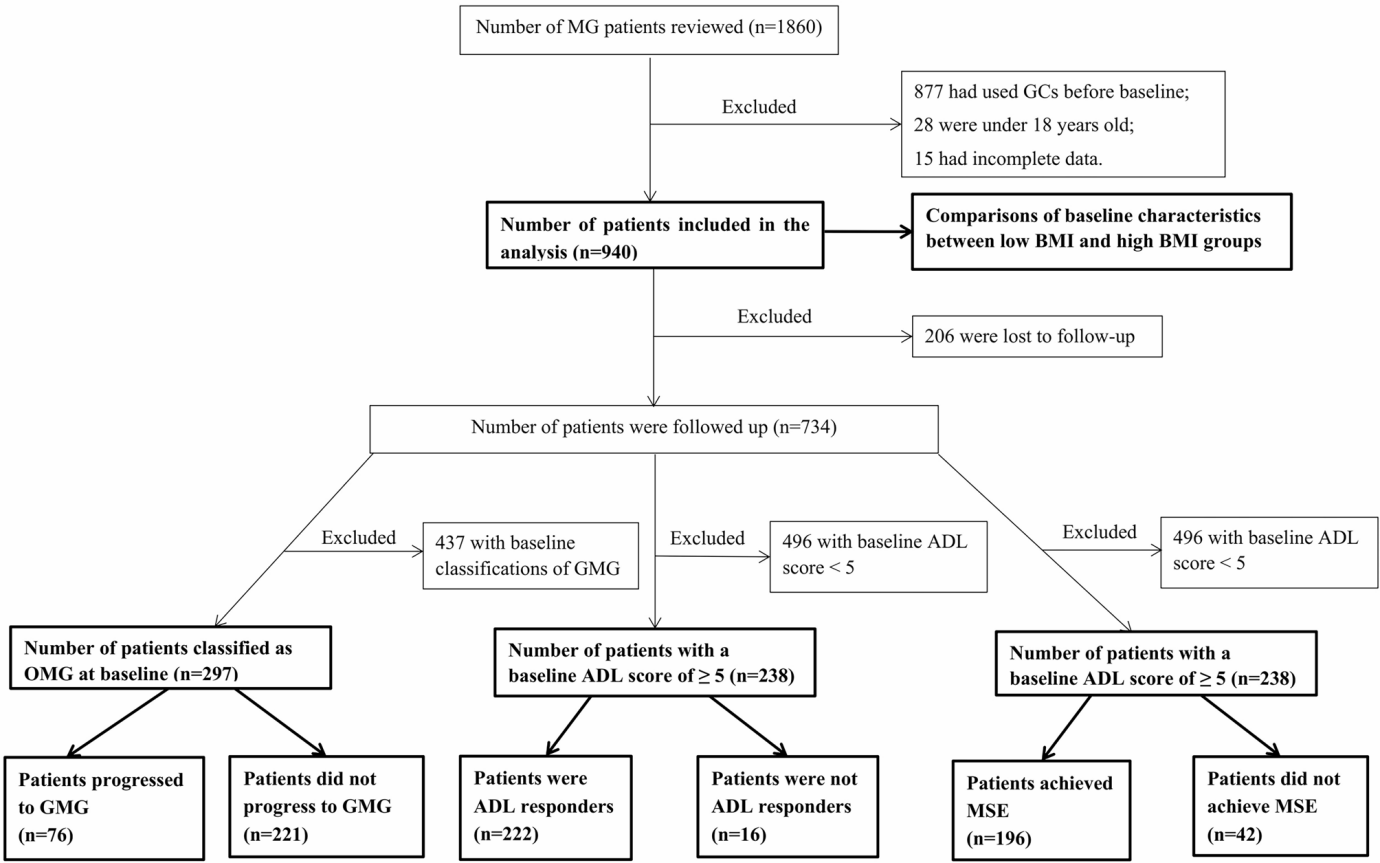
Study flowchart. Abbreviations: *MG*, myasthenia gravis; *GCs*, glucocorticoids; *BMI*, body mass index; *GMG*, generalized MG; *OMG*, ocular MG; *ADL*, activities of daily living; *MSE*, minimal symptom expression

Out of the 1860 patients with MG registered in our medical centers, 920 were excluded for the following reasons: 877 had used GCs before baseline, 28 were under 18 years old, and 15 had incomplete data. Consequently, 940 patients were included in the study (524 with low BMI and 416 with high BMI). The screening process for participants is depicted in Fig. 1.

### Demographic and clinical features

Table 1 presents the baseline demographic and clinical characteristics of the included patients. The cohort had a median age of 50.00 (34.00–62.00) years and consisted of 510/940 (54.26%) females. The median disease duration was 8.60 (2.60-35.25) months, and the median BMI was 23.44 (21.08–25.93) kg/m². AChR-IgG was detected in 634/806 (78.66%) patients. Ocular onset occurred in 615/940 (65.43%) patients, and 391/940 (41.60%) patients were classified as OMG according to the MGFA. Thymoma was present in 172/751 (22.90%) patients, and 218/735 (29.66%) patients had undergone thymectomy.

Compared to patients with a low BMI, those with a high BMI were significantly older (*p* < 0.001), had a lower proportion of female patients (*p* < 0.001), and experienced a shorter disease duration (*p* = 0.014). Additionally, the high BMI group exhibited higher rates of ocular onset (*p* < 0.001), OMG classification (*p* = 0.001), and AChR-IgG seropositivity (*p* = 0.007), along with a lower incidence of thymectomy (*p* = 0.027). Although the prevalence of thymoma was lower in the high BMI group, this difference was not statistically significant (*p* = 0.085). The baseline ADL scores were similar between the low and high BMI groups (*p* = 0.310). (Table 1)

### Long-term prognosis of patients

In the follow-up study, 206 patients were lost to follow-up, leaving 734 patients who were regularly followed up through face-to-face or telephone every 3 to 6 months. Over a median follow-up period of 33.00 (26.00–39.00) months, 552 (75.20%) patients received immunotherapy based on the individualized discretion of their treating physicians, with a higher proportion in the low BMI group compared to the high BMI group (*p* = 0.015). Among patients classified as OMG at baseline (*n* = 297), fewer in the low BMI group progressed to GMG compared to the high BMI group during the follow-up (*p* = 0.036). For patients with a baseline ADL score of ≥ 5 (*n* = 238), the proportion of ADL responders (*p* = 0.632) and those achieving MSE (*p* = 0.677) was similar between the low and high BMI groups. (Table 2)

**Fig. 2:**
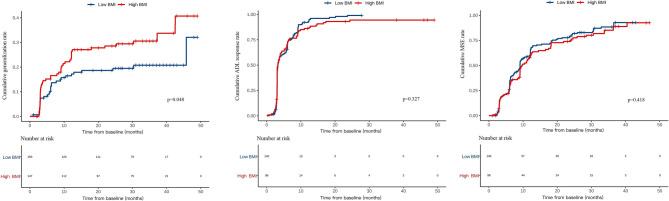
Kaplan–Meier curves showing the cumulative rates of generalization of ocular myasthenia gravis, ADL response, and MSE between different groups. Abbreviations: *BMI*, body mass index; *ADL*, activities of daily living; *MSE*, minimal symptom expression

The Kaplan–Meier curves illustrated the cumulative generalization rates of OMG, ADL response rates, and MSE rates between the low BMI and high BMI groups (Fig. 2). Patients with a high BMI showed a higher cumulative generalization rate compared to those with a low BMI (*p* = 0.048). However, no significant differences were found in cumulative ADL response rates (*p* = 0.327) and MSE rates (*p* = 0.418) between the low and high BMI groups.

To evaluate the impact of baseline BMI on long-term prognosis, Cox proportional hazards regression models were conducted, adjusting for age, sex, disease duration, MGFA classification, symptoms at onset, thymectomy, thymoma, AChR-IgG seropositivity, and immunotherapy. The results are summarized in Table 3. The analysis revealed that baseline BMI helped to predict the risk of progressing to GMG (HR 1.06; 95% CI 1.01–1.11; *p* = 0.026), with each additional 1 kg/m² increasing the risk of generalization by 6%. However, baseline BMI was not associated with the risk of ADL response (HR 0.99; 95% CI 0.95–1.04; *p* = 0.779) or MSE (HR 0.97; 95% CI 0.92–1.02; *p* = 0.240).

## Discussion

In China, the mean BMI in adults has been steadily increasing since the early 1980s. Globally, the prevalence of obesity in adults has almost tripled since 1975 [19]. Consequently, exploring the relationship between BMI and health is of great importance. Previous studies have shown that obesity is associated with various autoimmune diseases. In this large multicenter cohort study, we aimed to investigate how baseline BMI influences the clinical presentation and prognosis of MG. Our findings revealed that MG patients with a high BMI were older, had a shorter disease duration, and included fewer female patients. These patients also exhibited higher rates of ocular onset, OMG classification, and AChR-IgG seropositivity, along with a lower incidence of thymectomy. Furthermore, the Cox proportional hazards regression models indicated that a high baseline BMI was associated with a greater risk of OMG generalization, although it was not related to the risk of ADL response or MSE.

There is a strong link between obesity and various autoimmune disorders. Adipose tissue functions not just as a storage tissue but as an endocrine organ that produces numerous soluble mediators called adipokines, which are involved in metabolism, immunity, and inflammation. Increasing evidence shows that chronic, low-grade inflammation in visceral adipose tissue is associated with several immune-mediated disorders in obese individuals. In fact, pro-inflammatory and autoreactive antibodies, characteristic of autoimmune disorders, are found in greater numbers in both animal models and human subjects with obesity, likely due to CD40L signaling that enhances the production of inflammatory cytokines in adipocytes [20, 21]. In our current MG cohort, autoantibodies (AChR-IgG) were also more prevalent in patients with a high BMI.

Previous research on the impact of BMI on MG has yielded varying results. Chronic diseases commonly reduce patients’ quality of life, and MG is no exception. Several studies have examined the factors influencing quality of life in MG, revealing that, aside from high disease severity, patients with a high BMI experience a significantly diminished quality of life [10, 22]. Additionally, obesity worsens postoperative complications following thymectomy in MG patients [11, 13]. However, a recent study indicated that a high BMI might predict improved short-term (6 months) outcomes, likely due to the higher incidence of dual therapy (GCs and tacrolimus) in these patients [9]. In our current study, the median baseline ADL scores were similar between the low and high BMI groups, indicating comparable baseline disease severity. After a median follow-up of 33 months (similar for both BMI groups), baseline BMI was not associated with the risk of ADL response or MSE. However, a high BMI increased the risk of progression from OMG to GMG. In our cohort, fewer high BMI patients received immunotherapy during follow-up, which could influence the generalization of OMG. Nevertheless, in multivariable Cox proportional hazards regression models, which adjusted for immunotherapy and other potential confounders (age, sex, disease duration, thymectomy, thymoma, AChR-IgG seropositivity), the effect of high BMI on the generalization of OMG persisted. Preventing the progression from OMG to GMG is crucial for disease outcomes [2, 3]. Our findings suggest that weight loss might benefit MG patients.

A recent comprehensive national survey in China showed that the lowest mean BMI was among individuals aged 18–29 years. In 2004, men had a lower mean BMI than women, but by 2018, this pattern had reversed, with BMI increasing more rapidly in men than in women between 2004 and 2018 [19]. The distribution of BMI in our cohort was consistent with these findings, where MG patients with a high BMI were older and included fewer female patients. Additionally, GCs are commonly used as first-line immunotherapy for MG due to their rapid effectiveness, which is particularly crucial for patients with severe MG. However, weight gain is a common side effect of GCs [1]. Therefore, to eliminate the effect of medications on BMI, we excluded patients who had used GCs before baseline. In the current study, patients with a high BMI showed shorter disease duration and higher rates of ocular onset and OMG classification. The underlying reason for the higher incidence of OMG in obese patients remains unclear. Nevertheless, diet is not considered the main cause of OMG, since OMG patients do not experience generalized symptoms or difficulties with eating. In contrast, GMG patients may be involved of limb and axial muscles, as well as oropharyngeal and respiratory muscles, leading to fatigue and challenges with swallowing or breathing. These issues can result in insufficient food intake and malnutrition. The longer the disease lasts, the more significant the weight loss due to undereating. Consequently, patients with generalized symptoms and longer disease duration might be more prone to having a lower BMI.

In this nationwide multicenter study, trained research assistants and neurologists collected all data following standardized procedures. Patients were included consecutively and followed up regularly. However, our study has some limitations. Firstly, we did not investigate the exact dosages and timing of non-glucocorticoid immunotherapy, and which may be important for evaluating their role in the outcomes of MG patients. Nevertheless, all treatment physicians in our cohort were experienced neurological clinicians who had long been involved in the treatment of myasthenia gravis and were well trained before the study. Secondly, we assessed the impact of BMI only at baseline, despite the fact that BMI can change over time. Thirdly, we did not obtain precise body composition data, so relying solely on BMI might skew the results. Fourthly, testing for anti-lipoprotein-receptor-related protein 4 antibodies was not routinely conducted in double-seronegative (anti-AChR and anti-MuSK antibodies) cases, which may have resulted in missed diagnoses in a small number of instances. Future studies are needed to explore the impact of BMI changes on MG prognosis and their causal relationship.

**Table 1:** Baseline characteristics of MG patients according to BMI groups

	Total (*n* = 940)	Low BMI (*n* = 524)	High BMI (*n* = 416)	*p*-value
Female, n (%)	510(54.26)	336(64.12)	174(41.83)	< 0.001
Age, median(IQR), years	50.00 (34.00–62.00)	44.00 (29.00–58.00)	55.00 (44.75–64.25)	< 0.001
Disease duration, median(IQR), months	8.60(2.60- 35.25)	10.70 (2.88–37.20)	6.40 (2.48–26.98)	0.014
MGFA classification, n (%)				
Ocular MG (class I)	391(41.60)	194 (37.02)	197(47.36)	0.001
Generalized MG	549(58.40)	330 (62.98)	219(52.64)	
Class IIa	172(18.30)	98(18.70)	74(17.79)	0.719
Class IIb	227(24.15)	138(26.34)	89(21.39)	0.079
Class IIIa	46(4.89)	29(5.53)	17(4.09)	0.307
Class IIIb	61(6.49)	37(7.06)	24(5.77)	0.425
Class IVa	2(0.21)	1(0.19)	1(0.24)	0.999
Class IVb	12(1.28)	7(1.34)	5(1.20)	0.856
Class V	29(3.09)	20(3.82)	9(2.16)	0.145
Symptoms at onset, n(%)				< 0.001
Ocular	615(65.43)	316(60.31)	299 (71.88)	
Generalized	325(34.57)	208 (39.69)	117 (28.13)	
AChR-IgG serology, n (%)				0.007
Seropositivity	634(78.66)	341(75.11)	293 (83.24)	
Seronegativity	172(21.34)	113(24.89)	59(16.76)	
Missing	134	70	64	
Thymic status, n (%)				0.085
Thymoma	172(22.90)	107 (25.36)	65(19.76)	
Non-thymoma	579(77.10)	315 (74.64)	264(80.24)	
Missing	189	102	87	
Thymectomy, n(%)				0.027
Yes	218(29.66)	136(33.09)	82 (25.31)	
No	517(70.34)	275(66.91)	242(74.69)	
Missing	205	113	92	
Baseline ADL scores, median (IQR)	3.00(2.00–5.00)	3.00 (2.00–6.00)	3.00 (2.00,-5.00)	0.310

Abbreviations: MG, myasthenia gravis; BMI, body mass index; IQR, interquartile range; MGFA, Myasthenia Gravis Foundation of America; AChR, acetylcholine receptor; ADL, Activities of Daily Living. *P*-values for comparisons between groups were determined using Pearson’s chi-squared test or the Kruskal-Wallis rank sum test

**Table 2:** Characteristics of MG patients during the follow-up period

	Total (*n* = 734)	Low BMI (*n* = 412)	High BMI (*n* = 322)	*p*-value
Follow-up duration, median (IQR), months	33.00 (26.00–39.00)	32.00 (25.75-37.00)	35.00 (26.00–43.00)	0.083
Immunotherapy, n(%)	552(75.20)	324(78.64)	228(70.81)	0.015
Patients classified as ocular MG at baseline, n	297	150	147	—
Patients progressed to generalized MG, n (%)	76(25.59%)	30(20.00%)	46(31.29%)	0.036
Patients with a baseline ADL score of ≥ 5, n	238	140	98	—
ADL responders, n (%)	222(93.28%)	132(94.29%)	90(91.84%)	0.632
Patients achieving MSE, n (%)	196(82.35%)	117(83.57%)	79(80.61%)	0.677

Abbreviations: MG, myasthenia gravis; IQR, interquartile range; ADL, Activities of Daily Living; MSE, minimal symptom expression; BMI, body mass index. *P*-values for comparisons between groups were calculated using Pearson’s chi-squared test or the Kruskal-Wallis rank sum test

**Table 3:** Risk factors for the generalization of ocular MG, ADL response, and MSE in multivariable Cox proportional hazards regression models

Risk factors	Generalization of ocular MG			ADL response			MSE	
	Adjusted HR(95% CI)	*p*-value		Adjusted HR(95% CI)	*p*-value		Adjusted HR(95% CI)	*p*-value
BMI at baseline (per 1 kg/m^2^)	1.06(1.01–1.11)	0.026		0.99(0.95–1.04)	0.779		0.97(0.92–1.02)	0.240
Age (per year)	1.02(1.00-1.03)	0.070		0.99(0.98-1.00)	0.103		0.99(0.98-1.00)	0.018
Sex	1.43(0.87–2.37)	0.162		1.23(0.87–1.74	0.251		1.06(0.74–1.53)	0.750
Disease duration (per month)	1.00(1.00–1.00)	0.688		1.00(1.00–1.00)	0.171		1.00(0.99-1.00)	0.071
MGFA classification	—	—		1.64(1.05–2.56)	0.030		1.46(0.93–2.30)	0.102
Symptoms at onset	—	—		1.12(0.77–1.63)	0.539		1.40(0.93–2.10)	0.105
Thymectomy	2.22(1.04–4.76)	0.040		0.90(0.59–1.35)	0.598		0.95(0.63–1.44)	0.814
Thymoma	1.05(0.44–2.52)	0.904		1.01(0.65–1.57)	0.955		0.85(0.53–1.36)	0.498
AChR-IgG seropositivity	0.88(0.47–1.67)	0.704		1.30(0.80–2.13)	0.288		2.22(1.25–3.95)	0.007
Immunotherapy during follow-up	1.88(1.05–3.35)	0.033		1.58(1.05–2.36)	0.028		1.47(0.95–2.26)	0.080

Abbreviations: MG, myasthenia gravis; ADL, Activities of Daily Living; MSE, minimal symptom expression; BMI, body mass index; MGFA, Myasthenia Gravis Foundation of America; AChR, acetylcholine receptor; HR, Hazard Ratio; CI, Confidence Interval

## Conclusions

In summary, this study has demonstrated the impact of BMI on the clinical presentation and prognosis of MG. We found that a high baseline BMI was associated with an increased risk of OMG generalization, although it was not linked to the risk of ADL response or MSE.

## Data Availability

The data that support the findings of this study are included in this published article.

## References

[1] Gilhus NE, Tzartos S, Evoli A, Palace J, Burns TM, Verschuuren J. Myasthenia Gravis. Nat Rev Dis Primers. 2019;5:30. 31048702 10.1038/s41572-019-0079-y

[2] Guo RJ, Gao T, Ruan Z, et al. Risk factors for generalization in patients with ocular myasthenia gravis: A multicenter retrospective cohort study. Neurol Ther. 2022;11:73–86. 34729706 10.1007/s40120-021-00292-x PMC8857387

[3] Li F, Hotter B, Swierzy M, Ismail M, Meisel A, Rückert JC. Generalization after ocular onset in myasthenia gravis: a case series in Germany. J Neurol. 2018;265:2773–82. 30225725 10.1007/s00415-018-9056-8

[4] Winer S, Paltser G, Chan Y, et al. Obesity predisposes to Th17 bias. Eur J Immunol. 2009;39:2629–35. 19662632 10.1002/eji.200838893

[5] Tsigalou C, Vallianou N, Dalamaga M. Autoantibody production in obesity: is there evidence for a link between obesity and autoimmunity?? Curr Obes Rep. 2020;9:245–54. 32632847 10.1007/s13679-020-00397-8

[6] Luo W, Wang X, Kong L, Chen H, Shi Z, Zhou H. Initial BMI effects on clinical presentation and prognosis in neuromyelitis Optica spectrum disorder. Ann Clin Transl Neurol. 2023;10:1673–81. 37496188 10.1002/acn3.51857 PMC10502628

[7] Harpsøe MC, Basit S, Andersson M, et al. Body mass index and risk of autoimmune diseases: a study within the Danish National birth cohort. Int J Epidemiol. 2014;43:843–55. 24609069 10.1093/ije/dyu045

[8] Uzawa A, Kuwabara S, Suzuki S, et al. Roles of cytokines and T cells in the pathogenesis of myasthenia Gravis. Clin Exp Immunol. 2021;203:366–74. 33184844 10.1111/cei.13546 PMC7874834

[9] Liang F, Yin Z, Li Y et al. Constructing and validating a nomogram model for Short-Term prognosis of patients with AChR-Ab + GMG. Neurol Ther 2024. 10.1007/s40120-024-00590-0 PMC11136915 38427273

[10] Szczudlik P, Sobieszczuk E, Szyluk B, Lipowska M, Kubiszewska J, Kostera-Pruszczyk A. Determinants of quality of life in myasthenia Gravis patients. Front Neurol. 2020;11:553626. 33071942 10.3389/fneur.2020.553626 PMC7538807

[11] Liu XD, Shao MR, Sun L, Zhang L, Jia XS, Li WY. Influence of body mass index on postoperative complications after thymectomy in myasthenia Gravis patients. Oncotarget. 2017;8:94944–50. 29212280 10.18632/oncotarget.19189 PMC5706926

[12] Zhou Q, Yin W, Zhu J, et al. Risk factors associated with adverse pregnancy outcomes and postpartum exacerbation in women with myasthenia Gravis. Am J Reprod Immunol. 2022;88:e13641. 36305609 10.1111/aji.13641

[13] Leuzzi G, Meacci E, Cusumano G, et al. Thymectomy in myasthenia gravis: proposal for a predictive score of postoperative myasthenic crisis. Eur J Cardiothorac Surg. 2014;45:e76–88. discussion e88. 24525106 10.1093/ejcts/ezt641

[14] Gilhus NE. Myasthenia Gravis. N Engl J Med. 2016;375:2570–81. 28029925 10.1056/NEJMra1602678

[15] Wolfe GI, Herbelin L, Nations SP, Foster B, Bryan WW, Barohn RJ. Myasthenia Gravis activities of daily living profile. Neurology. 1999;52:1487–9. 10227640 10.1212/wnl.52.7.1487

[16] China NHCotFPCotPsRo. Criteria of weight for adults (WS/T 428–2013). 2013.

[17] Muppidi S, Silvestri NJ, Tan R, Riggs K, Leighton T, Phillips GA. Utilization of MG-ADL in myasthenia Gravis clinical research and care. Muscle Nerve. 2022;65:630–9. 34989427 10.1002/mus.27476 PMC9302997

[18] Vissing J, Jacob S, Fujita KP, O’Brien F, Howard JF. Minimal symptom expression’ in patients with acetylcholine receptor antibody-positive refractory generalized myasthenia Gravis treated with Eculizumab. J Neurol. 2020;267:1991–2001. 32189108 10.1007/s00415-020-09770-y PMC7320935

[19] Wang L, Zhou B, Zhao Z, et al. Body-mass index and obesity in urban and rural china: findings from consecutive nationally representative surveys during 2004-18. Lancet. 2021;398:53–63. 34217401 10.1016/S0140-6736(21)00798-4 PMC7617101

[20] Shoenfeld Y. Everything is autoimmune until proven otherwise. Clin Rev Allergy Immunol. 2013;45:149–51. 23907711 10.1007/s12016-013-8385-8

[21] Petta I, Fraussen J, Somers V, Kleinewietfeld M. Interrelation of diet, gut microbiome, and autoantibody production. Front Immunol. 2018;9:439. 29559977 10.3389/fimmu.2018.00439 PMC5845559

[22] Wilcke H, Glaubitz S, Kück F, et al. Female sex and overweight are associated with a lower quality of life in patients with myasthenia gravis: a single center cohort study. BMC Neurol. 2023;23:366. 37817097 10.1186/s12883-023-03406-0 PMC10563278

